# Understanding refugee and immigrant health literacy and beliefs toward antimicrobial resistance

**DOI:** 10.1017/ash.2023.383

**Published:** 2023-09-29

**Authors:** Joseph Ladines-Lim, Elizabeth Scruggs, Tessa Adzemovic, Rachel Croxton, Ron Romero, Michael Lukela, Preeti Mehrotra, Payal Patel

## Abstract

**Background:** Antimicrobial resistance (AMR) is a global health threat, particularly in refugee populations, due to challenges posed by migration. Little guidance has been provided by public health agencies regarding antimicrobial stewardship specific to this demographic. Studies have primarily focused on encampment areas abroad. We sought to better understand health literacy and beliefs regarding AMR in local refugee and immigrant populations in southeastern Michigan. **Methods:** From November 1, 2022 to March 10, 2023, we distributed an anonymous questionnaire to adult patients at four primary care clinics in Southeastern Michigan and made it available online. The questionnaire collected demographic information and used 5-point Likert scale responses regarding antibiotic use in children with symptoms of respiratory infection. We binarized the questions and responses to determine whether respondents provided the preferred response and added these to create an overall health literacy score, then used simple linear and multivariable linear regression modeling to identify demographic variables independently associated with the health literacy score. Chi-squared and Mann-Whitney tests were also performed where appropriate. **Results:** Immigrants and refugees/asylum-seekers from low or middle-income countries (group A, n = 109) were compared to native-born Americans and immigrants from high-income countries (group B, n = 171) with participants from 40 countries (Figure 1). Age distribution did not differ between groups, while group B had generally longer duration of living in the United States (Figure 2). Differences were found in other demographic categories except female gender, with group B reporting higher income, educational levels, and English ability (Figure 3). Simple linear regression revealed that all demographic variables except age significantly correlated with responses (Figure 4). Multivariable linear regression showed that female gender, educational level, and age correlated with greater health literacy, while being in group A trended towards significance with respect to correlating with lesser health literacy (Figure 5). **Conclusions:** Immigrants and refugees/asylum-seekers from LMICs demonstrated beliefs suggesting deficits in knowledge of AMR compared to native-born Americans and those from high-income countries, independent of other potentially confounding demographic characteristics. Female gender, educational level, and age independently correlated with greater health literacy. These results could inform future patient-centered antimicrobial stewardship educational interventions in certain target populations such as immigrants and refugees/asylum-seekers in the United States.

**Figure:**
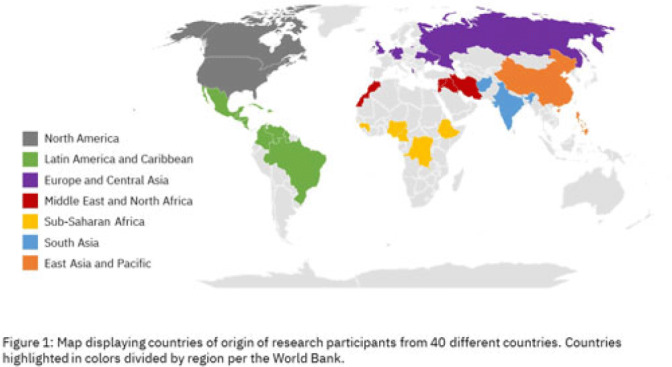

**Figure:**
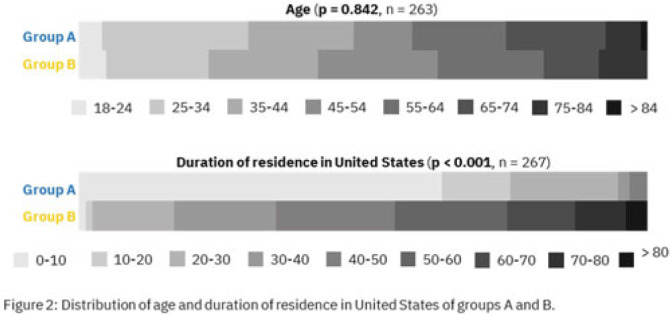

**Figure:**
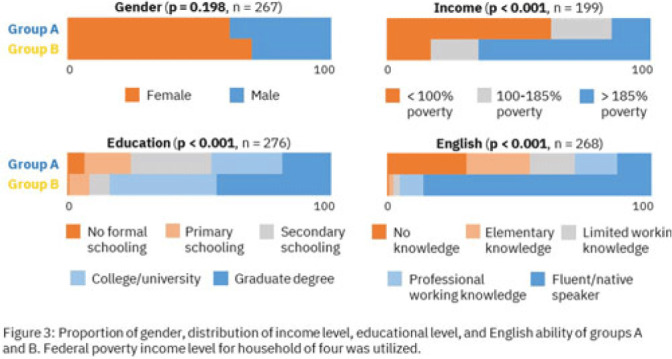

**Figure:**
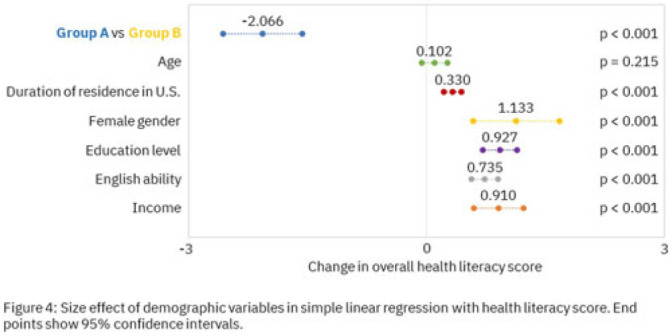

**Figure:**
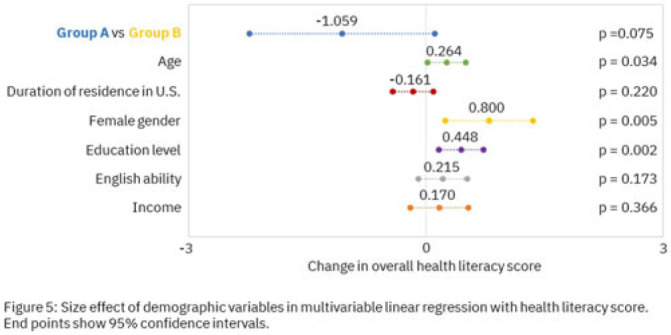

**Disclosures:** None

